# Cerebral Malaria: An Unusual Cause of Central Diabetes Insipidus

**DOI:** 10.1155/2016/2047410

**Published:** 2016-05-08

**Authors:** Resmi Premji, Nira Roopnarinesingh, Joshua Cohen, Sabyasachi Sen

**Affiliations:** Division of Endocrinology, Diabetes and Metabolism, George Washington University, Washington, DC 20037, USA

## Abstract

Central diabetes insipidus is an uncommon feature of malaria. A previously healthy 72-year-old man presented with fever, rigors, and altered mental status after a recent trip to Liberia, a country known for endemic falciparum malaria. Investigations confirmed plasmodium falciparum parasitemia. Within one week after admission, the serum sodium rose to 166 mEq/L and the urine output increased to 7 liters/day. Other labs were notable for a high serum osmolality, low urine osmolality, and low urine specific gravity. The hypernatremia did not respond to hypotonic fluids. Diabetes insipidus was suspected and parenteral desmopressin was started with a prompt decrease in urinary output and improvement in mental status. Additional testing showed normal anterior pituitary hormones. The desmopressin was eventually tapered off with complete resolution of symptoms. Central diabetes insipidus occurred likely as a result of obstruction of the neurohypophyseal microvasculature. Other endocrinopathies that have been reported with malaria include hyponatremia, adrenal insufficiency, hypothyroidism, hypocalcemia, hypophosphatemia, hyper-, and hypoglycemia, but none manifested in our patient. Though diabetes insipidus is a rare complication of malaria, clinicians need to be aware of this manifestation, as failure to do so may lead to fatality particularly if the patient is dehydrated.

## 1. Introduction

Malaria remains a major public health challenge. As per WHO, there were about 198 million cases of malaria in 2013 with an estimated 584,000 deaths. Cerebral malaria is defined as an unexplained coma in a patient with malarial parasitemia. This is a life-threatening complication of malaria. Cytoadherence and sequestration of red blood cells in the microvasculature are thought to be the key pathophysiology. Here we present the case report of a 72-year-old patient who developed an unusual complication of cerebral malaria and central diabetes insipidus followed by a review on endocrine complications associated with malaria.

## 2. Case Presentation

A previously healthy 72-year-old man presented with a four-day history of fever, rigors, back pain, and altered mental status. He had returned from a trip to Liberia, a country with endemic chloroquine-resistant falciparum malaria, two weeks prior to presentation. He took doxycycline chemoprophylaxis during the trip however discontinued it prematurely upon return.

Investigations confirmed plasmodium falciparum parasitemia (greater than 10%) with acute kidney injury. He was started on parenteral quinidine followed by artesunate and exchange transfusion. The serum sodium was 132 mEq/L at admission. A head CT scan at admission did not reveal any acute findings. A brain MRI done 2 months prior as work up of an unexplained syncope was within normal limits.

**Figure 1:**
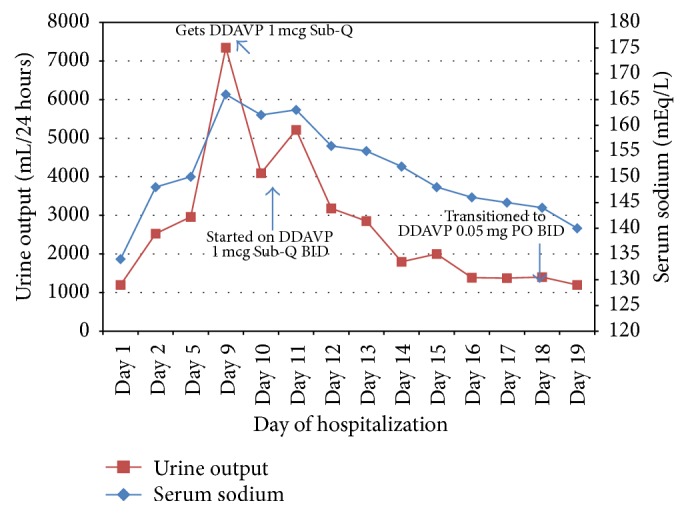
Graph of patient's serum sodium and urine output in response to desmopressin over hospital course. Sub-Q: subcutaneous; DDAVP: desmopressin; PO: per os; BID: twice daily.

Within a week after admission, there was rise in serum sodium and urine output as shown in Figure 1. Other studies were notable for high serum osmolality (354 mOsm/Kg), low urine osmolality (199 Osm/kg), and low urine specific gravity (1.010).

The hypernatremia did not respond to fluid therapy. Diabetes insipidus was suspected based on the above findings and parenteral desmopressin was started with a prompt decrease in serum sodium and urine output (Figure 1). This was accompanied by a significant improvement in mental status. Additional testing revealed morning cortisol 15.5 mcg/dL, free T4 1.3 ng/dL, and prolactin 34 ng/mL. These were done to exclude possibilities of adrenal and thyroid insufficiency. Acute kidney injury resolved with hydration and other electrolytes were found to be within normal limits as follows: calcium: 8.8 mg/dL, phosphorous: 3.2 mg/dL, and magnesium: 2.1 mg/dL. Fasting glucose ranged between 70 and 130 mg/dL during the hospital stay. Desmopressin was eventually changed to 0.05 mg twice daily orally on discharge and 0.05 mg at bedtime a week later. The serum sodium was 134 mEq/L on the day of discharge. Desmopressin was discontinued two weeks later with complete resolution of thirst and polyuria and serum sodium remained normal at 138 mEq/L during that time.

## 3. Discussion

We have described a patient who presented with cerebral malaria developing a very rare complication, central diabetes insipidus. The classic presentation of malaria with relapsing and remitting fevers occurs in only 50–70% of persons infected with plasmodium species [1] and the rest is accounted by atypical features.

### 3.1. Literature Review on Endocrine Abnormalities Associated with Malaria

Endocrine and metabolic abnormalities that have been reported in the literature with malaria include hyponatremia, diabetes insipidus, hypercortisolemia, adrenal insufficiency, primary and secondary hypothyroidism, hyper- and hypoglycemia, hyper- and hypokalemia, hypophosphatemia, hypocalcemia, and hypomagnesemia. Hyponatremia could result due to SIADH (syndrome of inappropriate antidiuretic hormone), cerebral salt wasting, or gastrointestinal and renal losses. Holst and his group [2] estimated antidiuretic hormone (ADH) concentrations along with serum and urine osmolality, serum, and urine sodium in 17 patients with falciparum malaria and found that the ADH concentrations were raised inappropriately in relation to the serum osmolality in 6 patients. The high ADH might be a marker of decrease in the effective plasma volume suggesting TNF-alpha induced endothelial damage and leakage. However, dehydration more than SIADH was suggested as the cause of hyponatremia in severe childhood malaria according to English and colleagues [3]. In their study 47 children with cerebral malaria were prospectively recruited and serial indices of fluid/electrolyte balance and renal function were monitored during admission. 21% of patients had pronounced hyponatremia and in comparison to the eunatremic group, hyponatremic children gained less weight, had higher urea concentrations, and were more acidotic consistent with dehydration.

Diabetes insipidus as occurred in our patient could have resulted due to obstruction of neurohypophyseal microvasculature by the malarial parasites. Mature forms of the parasite, including trophozoites and the meronts, sequester in the microvasculature; nonparasitized RBCs adhere to the parasitized RBCs (rosetting) and parasitized RBCs adhere among each other (agglutination). As the parasites grow within the RBCs, they become less deformable causing mechanical obstruction and impaired microvascular circulation. A variety of proinflammatory cytokines, including TNF-alpha increases the cytoadherence [4]. Only a few case reports have described diabetes insipidus as a complication of cerebral malaria. In a study conducted by Grimwade et al. [5], in 411 patients with malaria, 37 of 175 patients with severe malaria had polyuria, and 10 patients had low urine osmolality and high serum osmolality consistent with diabetes insipidus. Another case report by Schubert et al. [6] described central diabetes insipidus in a patient with cerebral malaria, which responded to desmopressin. The microvascular obstruction gradually resolves with treatment of the parasitemia and hence the requirement of desmopressin is temporary as illustrated in our case. The mild hyperprolactinemia noted was likely secondary to the temporary cessation of the regulatory dopamine impulses reaching the anterior pituitary.

Both cortisol excess and adrenal insufficiencies have been reported as a complication of malaria. In a study conducted by Wilson et al. [7] it was shown that, in persons infected with malaria, there is inappropriate suppression of cortisol after one milligram dexamethasone suppression test. They proposed that this could be possibly due to proinflammatory cytokines activating the adrenal cortex in order to protect from complications such as hypoglycemia associated with the infection. On the other hand, Davis et al. [8] in a study of 9 patients with complicated malaria found that the rise in ACTH and cortisol after CRH stimulation was blunted. The primary and secondary adrenal insufficiency may be attenuated by increased circulating IL-6 and impaired cortisol metabolism. Authors also suggested that if hypoglycemia was present and/or if cortisol levels were within or below the normal range, stress dose steroids should be considered. Secondary adrenal insufficiency and central hypothyroidism leading to delayed anesthetic recovery have been reported in a patient with history of cerebral malaria [9]. Another interesting feature described in the literature includes cortisolemia leading to loss of immunity in pregnant women infected with falciparum malaria. In this study Adam et al. investigated 50 consecutive women with uncomplicated falciparum malaria and observed a positive correlation between cortisol levels and parasite count (*r* = 0.332, *P* = 0.02) [10]. In our patient, there was no evidence of either primary or secondary adrenal insufficiency depicted by normal fasting morning cortisol of 15.5 mcg/dL and normal blood pressure throughout his hospital stay.

Thyroid abnormalities previously published include primary and secondary hypothyroidism [11]. These changes may be an adaptation to the accelerated catabolism when infected with malaria; however, the role of thyroid replacement in such patients is uncertain. Wartofsky et al. [12] studied thyroid function in healthy volunteers who were inoculated with malaria; they found an initial decrease followed by a rebound rise in thyroid hormone release. Our patient had normal thyroid axis as illustrated by normal free T4 of 1.3 ng/dL.

Thien and coworkers studied gluconeogenesis in different populations of individuals affected with falciparum malaria to various degrees of severity and found an increased rate of gluconeogenesis in both complicated and uncomplicated cases. This finding was additionally supported by the presence of low levels of the gluconeogenic amino acid precursor, glutamine. The authors commented that prolonged fasting is an important risk factor for hypoglycemia and full recognition of this complication is especially critical in the most susceptible population, children and pregnant women [13]. Another study by Kawo and colleagues did not find any significant difference in the frequency of hypoglycemia between malarial and control patients (5.2% versus 11.2%) nor between the comatose (11.1% versus 18.8%) and conscious malarial and control (1.6% versus 7.0%) subgroups [14]. Similar to the Thien et al. study, the authors emphasized hypoglycemia as a consequence of prolonged fasting resulting from glycogen depletion. Hyperglycemia could also occur because of associated sepsis or due to the stress response with increased counterregulatory hormones [15, 16]. Our patient was, however, euglycemic throughout the course of hospital stay.

Hyperkalemia associated with severe malaria is thought to be due to a combination of acute renal failure and lactic acidosis. Increased extra cellular release of potassium could also occur in the context of hemolysis and rhabdomyolysis. Our patient, although presented with acute kidney injury, did not develop hyperkalemia and there was a prompt decrease in creatinine with hydration. Hypokalemia had been described in up to 40% of children [17] with severe malaria by Maitland et al. within a few hours of hospitalization. The authors suggested that it could be due to rapid correction of acidosis leading to intracellular shift of potassium and/because of renal potassium loss. A lower renal threshold for phosphorus could lead to hypophosphatemia. Screening for hypophosphatemia was especially important in our patient as low phosphorous can further worsen the neuro hypophyseal perfusion through platelet dysfunction and decreased 2,3-diphosphoglycerate. The main reason for hypocalcemia cited in the literature is a blunted response of parathyroid hormone to hypocalcemia, also called “sick euparathyroid state” [18]. Mild asymptomatic hypomagnesemia is also known to occur with malaria and this in turn alters the set point of parathyroid hormone release with hypocalcemia. Throughout the hospital stay, our patient had normal potassium, phosphorous calcium, and magnesium levels.

In summary, central diabetes insipidus is a rare and atypical complication of malaria. Although uncommon, clinicians need to be aware of this manifestation since early diagnosis prevents serious outcomes. Particularly in hot, humid, and arid climates of sub-Saharan Africa and Indian subcontinent where parasitic infection and dehydration are common, dehydration compounded by diabetes insipidus can even lead to death if left unrecognized. Our patient did not have other metabolic or hormonal abnormalities that have been reported in the literature including hyponatremia, adrenal insufficiency, cortisol excess, thyroid dysfunction, hyper/hypoglycemia, or other electrolyte imbalances.

In our patient the diabetes insipidus symptoms lasted for around four weeks and desmopressin was eventually tapered off. It is important to carefully monitor other anterior pituitary hormone status along with electrolytes while caring for a patient with acute onset of central diabetes insipidus.

## References

[1] Krause P. J., Behrman R. E., Kliegman R. M., Jenson H. B. (2007). Malaria (Plasmodium). *Nelson Textbook of Pediatrics*.

[2] Holst F. G., Hemmer C. J., Kern P., Dietrich M. (1994). Inappropriate secretion of antidiuretic hormone and hyponatremia in severe falciparum malaria. *The American Journal of Tropical Medicine and Hygiene*.

[3] English M. C., Waruiru C., Lightowler C., Murphy S. A., Kirigha G., Marsh K. (1996). Hyponatraemia and dehydration in severe malaria. *Archives of Disease in Childhood*.

[4] Gimenez F., Barraud de Lagerie S. B., Fernandez C., Pino P., Mazier D. (2003). Tumor necrosis factor *α* in the pathogenesis of cerebral malaria. *Cellular and Molecular Life Sciences*.

[5] Grimwade K., French N., Mthembu D., Gilks C. (2004). Polyuria in association with *Plasmodium falciparum* malaria in a region of unstable transmission. *Transactions of the Royal Society of Tropical Medicine and Hygiene*.

[6] Schubert S., Achenbach H., Engelmann L., Borte G., Stumvoll M., Koch C. A. (2006). Central diabetes insipidus in a patient with malaria tropica. *Journal of Endocrinological Investigation*.

[7] Wilson M., Davis T. M. E., Binh T. Q., Long T. T. A., Danh P. T., Robertson K. (2001). Pituitary-adrenal function in uncomplicated falciparum malaria. *Southeast Asian Journal of Tropical Medicine and Public Health*.

[8] Davis T. M., Li T. A., Tran Q. B. (1997). The hypothalamic-pituitary adrenocortical axis in severe falciparum malaria: effects of cytokines. *The Journal of Clinical Endocrinology & Metabolism*.

[9] Selvaraj V. (2015). Hypopituitarism: a rare sequel of cerebral malaria—presenting as delayed awakening from general anesthesia. *Anesthesia: Essays and Researches*.

[10] Adam I., Nour B. Y., Almahi W. A., Omer E. S. M., Ali N. I. (2007). Malaria susceptibility and cortisol levels in pregnant women of eastern Sudan. *International Journal of Gynecology and Obstetrics*.

[11] Davis T. M. E., Supanaranond W., Pukrittayakamee S. (1990). The pituitary-thyroid axis in severe falciparum malaria: evidence for depressed thyrotroph and thyroid gland function. *Transactions of the Royal Society of Tropical Medicine and Hygiene*.

[12] Wartofsky L., Burman K. D., Dimond R. C., Noel G. L., Frantz A. G., Earll J. M. (1977). Studies on the nature of thyroidal suppression during acute falciparum malaria: Integrity of pituitary response to TRH and alterations in serum T_3_ and reverse T_3_. *The Journal of Clinical Endocrinology & Metabolism*.

[13] Thien H. V., Kager P. A., Sauerwein H. P. (2006). Hypoglycemia in falciparum malaria: is fasting an unrecognized and insufficiently emphasized risk factor?. *Trends in Parasitology*.

[14] Kawo N. G., Msengi A. E., Swai A. B. M., Chuwa L. M., Alberti K. G. M. M., McLarty D. G. (1990). Specificity of hypoglycaemia for cerebral malaria in children. *The Lancet*.

[15] Tombe M., Bhatt K. M., Obel A. O. (1993). Clinical surprises and challenges of severe malaria at Kenyatta National Hospital, Kenya. *East African Medical Journal*.

[16] Dass R., Barman H., Duwarah S. G., Deka N. M., Jain P., Choudhury V. (2010). Unusual presentations of malaria in children: an experience from a tertiary care centre in North East India. *Indian Journal of Pediatrics*.

[17] Maitland K., Pamba A., Newton C. R. J. C., Lowe B., Levin M. (2004). Hypokalemia in children with severe falciparum malaria. *Pediatric Critical Care Medicine*.

[18] Davis T. M., Li G. Q., Guo X. B., Spencer J. L., St John A. (1993). Serum ionized calcium, serum and intracellular phosphate, and serum parathormone concentrations in acute malaria. *Transactions of the Royal Society of Tropical Medicine and Hygiene*.

